# The Genome-Wide Identification and Expression Profiling of the HSF Gene Family in *Ganoderma lucidum* Under Temperature Stress

**DOI:** 10.3390/genes17040473

**Published:** 2026-04-17

**Authors:** Jinyu Hu, Yihong Li, Shaohua Wu, Liwei Liu, Jiawei Zhou, Wei Li, Rui Zhang, Zongsuo Liang, Dongfeng Yang, Zongqi Yang

**Affiliations:** Zhejiang Province Key Laboratory of Plant Secondary Metabolism and Regulation, College of Life Sciences and Medicine, Zhejiang Sci-Tech University, Hangzhou 310018, China

**Keywords:** *Ganoderma lucidum*, HSF, expression patterns, genome-wide identification, bioinformatics

## Abstract

Objective: In this study, the heat shock transcription factor (HSF) gene family in *Ganoderma lucidum* was systematically characterized. Using genomic and transcriptomic data, we identified HSF family members and investigated their expression patterns under temperature stress and their potential regulatory roles in triterpenoid biosynthesis. Methods: A genome-wide identification of *HSF* genes in *G. lucidum* was performed using bioinformatic approaches. A phylogenetic tree was constructed, and conserved motifs, gene structures, and protein tertiary structures were predicted. The relative expression levels of *HSF* genes and key mevalonate (MVA) pathway enzyme genes were examined by a quantitative real-time reverse transcription polymerase chain reaction (qRT-PCR) in mycelia subjected to temperature stress. Total triterpenoid content in fermented mycelia under temperature stress was determined using the vanillin–glacial acetic acid method. Results: Eight *HSF* family members (*GlHSF1*–*GlHSF8*) were identified in *G. lucidum*. Phylogenetic analysis revealed that GlHSF proteins were closely related to *PoHSF* from *Pleurotus ostreatus*. Transcriptomic analysis showed that *HSF* genes exhibited relatively high expression levels during the mature stage while being barely expressed during the mycelial stage. Under heat stress (42 °C), most *GlHSF* genes peaked at 18 h, with *GlHSF2* showing the most pronounced response (approximately 13-fold upregulation). Downstream MVA pathway genes, including *IDI*, *PMK*, and *MVD*, were significantly upregulated at 24 h, whereas the upstream rate-limiting enzyme gene *HMGR* was continuously suppressed. Despite *HMGR* suppression, total triterpenoid content did not decrease significantly, likely due to the activation of downstream genes. Under cold stress (14 °C), the expression of most *GlHSF* and MVA pathway genes decreased, accompanied by a significant reduction in total triterpenoid content. Conclusions: The *HSF* gene family was identified in the *G. lucidum* genome. Based on expression analysis, *GlHSF2* showed the strongest response under heat stress, and its expression peak was correlated with the sequential activation of downstream genes in the MVA pathway. This suggests that *GlHSF2* acts as a potential key regulatory node, differentially regulating upstream and downstream MVA pathway genes to influence triterpenoid biosynthesis under heat stress. These findings provide a theoretical basis for future research on the biological functions of *GlHSF* homeostasis.

## 1. Introduction

*Ganoderma lucidum* is a traditional precious medicinal fungus that is both edible and medicinal in China. However, the *HSF* gene family in *G. lucidum* has not yet been systematically characterized, and its expression patterns under temperature stress and regulatory relationship with triterpenoid biosynthesis remain unclear. Therefore, the main objectives of this study were to: (1) identify *HSF* family members at the genome-wide level and analyze their physicochemical properties, phylogeny, gene structures, conserved motifs, and promoter cis-acting elements; (2) characterize the spatiotemporal expression patterns of *GlHSF* genes based on transcriptomic data from six major developmental stages (mycelium, primordium, cap opening, maturity, fruiting body, and spore powder); and (3) investigate the association between *GlHSF* expression and the expression of key mevalonate (MVA) pathway enzyme genes as well as triterpenoid accumulation under temperature stress.

*G. lucidum* has a long history of application in the traditional medical systems of China and southeastern Asian countries [1]. According to the *Shennong Bencao Jing* (*Shennong’s Classic of Materia Medica*), *G. lucidum* is classified as a “superior herb”—a category for non-toxic substances that are beneficial for reinforcing vital energy and consolidating the constitution, thus earning its reputation as a “miraculous medicine” [2]. *G. lucidum* is rich in bioactive components such as polysaccharides, triterpenoids, and sterols, with ganoderma triterpenoids exhibiting anti-tumor [3], anti-carcinogenic [4], hepatoprotective [5], and antioxidant [6] activities. The completion of the genome sequencing of *G. lucidum* has facilitated functional genomic research. For example, Li et al. [7] identified *Zn_2_Cys_6__61* as a key positive regulator of ganoderic acid synthesis through omics analysis; Li et al. [8] reported that *GlbHLH* members exhibit a dual regulatory role in the upstream pathway of ganoderic acid biosynthesis; Wang et al. [9] found that *GlPRMT5* negatively regulates the biosynthesis of bioactive metabolites. Abiotic stresses, particularly temperature stress, severely inhibit mycelial growth and ganoderic acid biosynthesis [10], but the molecular mechanisms remain poorly understood.

HSFs are highly conserved core transcription factors that maintain protein homeostasis and counteract thermal and oxidative stresses [11]. The DNA-binding domain (DBD) of HSF specifically recognizes heat shock elements (HSEs) with the conserved motif nGAAn or nTTCnnGAAnnTTCn [11] and adopts a winged helix–turn–helix structure [11,12]. The DBD, hydrophobic heptad repeat (HR-A/B), and exon–intron boundaries define HSF phylogeny [13]. The HSF protein was first identified in *Drosophila melanogaster* in 1984 [14]. Subsequently, in 1988, a heat-inducible, sequence-specific DNA-binding protein was identified in yeast, leading to the cloning of *HSF* and the discovery of its temperature-dependent phosphorylation [15]. In 1993, the monomer-to-trimer conversion was uncovered as a key activation mechanism [16]. Upon heat stress, HSF monomers trimerize, translocate to the nucleus, bind to target sequences, and activate transcription. After cellular homeostasis is restored, negative feedback returns HSF to its monomeric state. In fungi, HSF maintains homeostasis by regulating the expression of molecular chaperones such as heat shock proteins (HSPs) [17], and the *HSF* gene family has been identified and its expression patterns analyzed in model fungi like *Saccharomyces cerevisiae* [15,18].

*G. lucidum* triterpenoids, which are primarily lanostane-type triterpenoids, represent one of the most abundant and pharmacologically active constituents of *G. lucidum* [19]. The key enzymes in the MVA [20] pathway by which *G. lucidum* produces ganoderic acids are *AACT* [21], *LS* [22], *HMGR* [23], *HMGS* [24], *MVD* [25], *IDI* [26], *FPS* [27], and *SQS* [28]. Heat stress often upregulates the MVA pathway by activating specific signaling cascades, thereby promoting the synthesis of secondary metabolites, including triterpenoids with antioxidant or membrane-stabilizing functions [10,29,30,31].

The main findings of this study are as follows: Eight *HSF* family members were identified. Under heat stress (42 °C), most *GlHSF* genes reached peak expression at 18 h, with *GlHSF2* showing the most pronounced upregulation (approximately 13-fold). Downstream MVA pathway genes were significantly upregulated at 24 h, whereas the upstream rate-limiting enzyme gene *HMGR* was continuously suppressed. These results suggest that *GlHSF2* is a candidate core responsive factor under heat stress, and its expression peak is temporally correlated with the activation of downstream MVA pathway genes, implying a possible role for GlHSF2 in the differential regulation of MVA pathway genes and, consequently, in triterpenoid biosynthesis. This study provides a theoretical basis and data support for the further functional analysis of *GlHSF* under temperature stress.

## 2. Materials and Reagents

### 2.1. Materials

The *G. lucidum* strain Xianzhi No. 1 (Zhejiang (Non-major) Approved Mushroom Variety 2009003) was obtained from the Zhejiang Province Key Laboratory of Plant Secondary Metabolism and Regulation. Three *G. lucidum* strains with consistent growth status were selected in this study and cultured independently as biological replicates (*n* = 3). Samples were collected at six distinct developmental stages (Figure S1): mycelium, primordium, cap opening, maturity, fruiting body, and sporulation (spore powder). One sample was collected from each strain, yielding a total of three independent biological replicates at each developmental stage. Samples from different growth stages were immediately frozen in liquid nitrogen and subsequently stored at −80 °C until further analysis. For subsequent experiments, *G. lucidum* mycelia were grown on potato dextrose agar (PDA) medium at 28 °C in the dark.

### 2.2. Reagents

Polysaccharides and Polyphenols Plant RNA Extraction Kit (RC411, Vazyme Biotech, Nanjing, China); EvoM-MLV Reverse Transcription Kit for qPCR (AG11728, Accurate Biotechnology, Hangzhou, China); SYBR^®^ Green Pro Taq HS Premix qPCR Kit (AG11718, Accurate Biotechnology); oleanolic acid standard (CAS: 508-02-1, Shanghai Yuanye Bio-Technology, Shanghai, China); vanillin (CAS: 121-33-5, TCI, Tokyo, Japan); and glacial acetic acid, ethyl acetate, and perchloric acid (analytical grade). Primers were synthesized by Tsingke Biotechnology (Beijing, China).

### 2.3. Culture Media

PDA medium (PM0520) was obtained from Coolaber Technology (Beijing, China).

Liquid seed medium: 35 g/L glucose, 5 g/L tryptone, 2.5 g/L yeast extract, 0.5 g/L MgSO_4_·7H_2_O, 1 g/L KH_2_PO_4_, and 0.05 g/L vitamin B_1_.

## 3. Methods

### 3.1. Identification and Physicochemical Characterization of GlHSF Genes

The genome-wide data of *G. lucidum* (GCA_000271565.1) [32] were retrieved from the NCBI database (https://www.ncbi.nlm.nih.gov; version 268.0, August 2025; accessed on 9 December 2025) [33]. The hidden Markov model (HMM) profile of the HSF transcription factor family (PF00096) was obtained from the Pfam database (http://pfam.xfam.org/; version 37.4, January 2025; accessed on 9 December 2025) [34]. Candidate genes containing the HSF domain were initially screened from the *G. lucidum* genome using the *hmmsearch* program from the HMMER software suite (version 3.3.2, November 2020) [35] with an E-value threshold of ≤0.01. Subsequently, BLASTP (version 2.17.0, July 2025) [36] searches against the NCBI database were performed to identify additional candidate genes by reverse-deducing gene sequences from protein sequences, using an E-value cut-off of ≤1 × 10^−5^. The genes identified by both methods were retained as candidate *HSF* genes. The molecular weight (MW) and theoretical isoelectric point (pI) of the candidate proteins were calculated using the *Peptides* package [37] in R software (version 4.5.2, October 2025). The subcellular localization of the *GlHSF* family members was predicted using the WoLF PSORT online tool (https://wolfpsort.hgc.jp/; version 0.2, September 2006; accessed on 10 December 2025) [38].

### 3.2. Chromosomal Localization and Phylogenetic Analysis of GlHSF Genes

The genomic locations of *GlHSF* genes were visualized using TBtools (version 1.130, July 2023) [39] software based on the *G. lucidum* genome assembly and structural annotation files. A chromosomal distribution map of *GlHSF* genes was generated by integrating their genomic positions with corresponding annotation information. For phylogenetic analysis, HSF protein sequences from *Pleurotus ostreatus*, *S. cerevisiae*, *Schizosaccharomyces pombe*, and other fungal species were retrieved from the NCBI and Pfam databases. These sequences, together with the identified GlHSF protein sequences from *G. lucidum*, were aligned using the Muscle program (version 3.8.31, August 2016) [40]. A maximum likelihood (ML) phylogenetic tree was constructed using IQ-TREE (version 2.1.3, June 2021) [41] with 1000 bootstrap replicates. The resulting phylogenetic tree was visualized and refined using the online tool iTOL (Interactive Tree of Life, https://itol.embl.de/; accessed on 12 December 2025) [42].

### 3.3. Sequence Analysis and Protein Structure Prediction of GlHSF Genes

The conserved motifs of the GlHSF proteins were predicted using the MEME online suite (http://meme-suite.org/index.html; version 5.5.1, February 2023; accessed on 12 December 2025) [43] with the following parameters: the number of motifs to identify was set to 10, and all other parameters were kept at their default values. A gene structure analysis of *GlHSF* genes was performed using the GSDS 2.0 (Gene Structure Display Server, http://gsds.gao-lab.org/; version 2.0, April 2015) [44] online tool. The three-dimensional (3D) structures of GlHSF proteins were predicted using the AlphaFold 3 [45] server (https://alphafoldserver.com; version 3, May 2024; accessed on 15 December 2025). For cis-acting element analysis, the 2000 bp upstream promoter sequences of *GlHSF* genes were extracted using TBtools software (version 1, June 2020). These sequences were then submitted to the PlantCARE [46] online platform (http://bioinformatics.psb.ugent.be/webtools/plantcare/html/; version 1.0, January 2002; accessed on 15 December 2025) for the prediction and analysis of cis-acting regulatory elements. All results obtained from the above analyses were visualized using TBtools software.

### 3.4. Expression Profile Analysis of GlHSF Genes at Different Developmental Stages of Ganoderma lucidum

Raw transcriptomic data from six developmental stages of *G. lucidum* (mycelium, primordium, cap opening, maturity, fruiting body, and sporulation) were processed using Fastp (version 0.21.0, February 2021) [47] for quality control and read trimming.

(1) Data quality control: The raw image data generated by high-throughput sequencing were converted into raw sequencing reads through base calling. Subsequently, Fastp was used to filter the raw data by removing reads shorter than 30 bp, contaminant sequences, low-quality reads, and reads with an excessively high proportion of ambiguous bases (N), thereby generating high-quality clean data. FastQC (version 0.11.9, March 2019) was then applied to evaluate the quality of the filtered clean data. (2) Alignment to the reference genome: Clean reads were aligned to the *G. lucidum* reference genome [48] using STAR (version 2.7.9a, October 2020) [49], and alignment statistics were calculated. (3) Gene expression quantification: featureCounts (version 1.5.0-p3, November 2014) [50] was used to count the reads mapped to each gene. The expression level of each gene was then calculated as FPKM (Fragments Per Kilobase of transcript per Million mapped reads). (4) Differential expression analysis: Differential expression analysis was performed using DESeq2 (version 1.46.0, October 2023) [51]. Low-expression genes were filtered based on CPM (counts per million) values, and the read count data were normalized. The base mean for each sample group was calculated, and the fold change was determined. Dispersion values were estimated using a negative binomial distribution model, followed by *p*-value calculation and Benjamini–Hochberg correction to obtain q-values. (5) Enrichment analysis: GO enrichment analysis and KEGG enrichment analysis were performed on the differentially expressed genes using topGO (version 2.56.0, October 2023) [52] and clusterProfiler (version 3.14.3, October 2020) [53], respectively.

Subsequently, the RSEM (version 1.3.3, October 2025) [54] and Trinity (version 2.15.2, May 2025) [55] software packages were used in combination to generate the expression matrix of *GlHSF* family genes. The resulting count data were normalized within samples using the TMM (trimmed mean of M-values) [56] method. A heatmap of *GlHSF* gene expression levels was then generated using the R programming language.

### 3.5. Expression Analysis of GlHSF and Key MVA Pathway Enzyme Genes in Response to Temperature Stress by qRT-PCR

Mycelial plugs were excised from vigorously growing colonies on the same plate and inoculated onto fresh potato dextrose agar (PDA) medium. The cultures were incubated at 28 °C in the dark until the mycelia covered approximately two-thirds of the plate. Subsequently, for each temperature treatment (14 °C, 28 °C, and 42 °C), three independent biological replicates (each from a separate mycelial culture) were prepared. Mycelia were harvested at 0, 6, 12, 18, and 24 h after temperature treatment.

Total RNA was extracted from the stressed mycelia using an RNA extraction kit and reverse-transcribed into cDNA using a reverse transcription kit. The expression levels of *GlHSF* family genes and key enzyme genes in the MVA pathway were quantified by qRT-PCR. The primers used are listed in Table 1. The *G. lucidum 18S rRNA* gene was used as an internal reference; the reaction system consisted of TB Green Premix Ex Taq (with ROX) 5.0 μL, PCR Forward Primer 0.2 μL, PCR Reverse Primer 0.2 μL, cDNA 1.0 μL, and ddH_2_O 3.6 μL. The PCR program was as follows: 95 °C for 30 s, followed by 40 cycles of 95 °C for 10 s and 60 °C for 34 s. All primers used for qPCR were designed to span exon–exon junctions where possible and validated by melting curve analysis to ensure a single specific amplicon. Standard curves were generated using serial dilutions of pooled cDNA to calculate amplification efficiency (E) for each primer pair according to the formula E = (10^(−1/slope) − 1) × 100%. Primer pairs exhibiting amplification efficiencies between 90% and 110% and a correlation coefficient (R^2^) greater than 0.99 were accepted. Each qPCR was performed in three technical replicates per biological replicate. The mean cycle threshold (Ct) of the technical replicates was used for subsequent calculations. Relative gene expression levels were calculated using the 2^−ΔΔCt^ method. Graphs were generated using GraphPad Prism 10 software, and a statistical analysis of relative expression levels was performed by an analysis of variance (ANOVA) using SPSS software (version 31.0.0, June 2025); differences were considered statistically significant at *p* < 0.05.

### 3.6. Determination of Total Triterpenoid Content in Ganoderma lucidum Mycelia Under Temperature Stress

Mycelial plugs excised from a vigorously growing colony were inoculated into primary liquid seed medium and incubated at 28 °C with shaking at 120 rpm for 7 days. Then, 2 mL of the primary culture was transferred into nine separate secondary liquid medium flasks (three independent biological replicates for each temperature condition) and incubated under the same conditions for an additional 3 days. After three days, the nine flasks were divided into three groups (three flasks per group) and subjected to static incubation at 14 °C, 28 °C, or 42 °C for 24 h. Subsequently, all flasks were transferred back to 28 °C for static incubation until a mycelial pellicle formed. After 10 days of static fermentation, the mycelial pellicles were harvested individually from each flask. Total triterpenoid content was subsequently determined using the vanillin–glacial acetic acid method [57] as described below.

An accurately weighed 1 mg of oleanolic acid reference standard was placed into a foil-wrapped glass test tube and dissolved in anhydrous ethanol, and the volume was adjusted to 10 mL to prepare a standard stock solution (0.1 mg·mL^−1^). Aliquots of 0, 0.2, 0.4, 0.6, 0.8, and 1.0 mL of the stock solution were transferred into separate 10 mL glass test tubes. The solvent was evaporated to dryness in a boiling water bath (100 °C). After cooling to room temperature, 0.1 mL of 5% (*w*/*v*) vanillin–glacial acetic acid solution and 0.4 mL of perchloric acid were added to each tube and mixed thoroughly. The mixtures were incubated in a 70 °C water bath for 15 min, followed by cooling in an ice bath for 5 min to terminate the reaction. Subsequently, 2 mL of ethyl acetate was added and mixed well. The absorbance of each solution was measured at 548 nm. A standard curve was constructed by plotting the absorbance (Y) against the mass of oleanolic acid (X, mg).

The linear regression equation of the standard curve was as follows:

Y = 13.477X + 0.0562 (R^2^ = 0.9991), where Y represents the absorbance and X represents the mass of oleanolic acid (mg).

Lyophilized mycelial samples were ground into a fine powder using a liquid nitrogen grinding mill. An accurately weighed amount of 25 mg of the powder was transferred into a 2 mL centrifuge tube, and 1.5 mL of 95% ethanol was added. The mixture was extracted at room temperature for 2 h with vortexing every 30 min, followed by ultrasonic extraction for 30 min. After centrifugation at 4000 rpm for 15 min, 200 µL of the supernatant was transferred into a glass test tube (three replicates per sample). The solvent was evaporated to dryness in a boiling water bath (100 °C). The subsequent color development and absorbance measurement steps were performed as described for the standard curve method. Total triterpenoid content in the samples was calculated based on the regression equation obtained from the standard curve.

The total triterpenoid content was calculated using the following formula:

Total triterpenoid content (mg/100 mg cell dry weight) = [(Y − 0.0562)/13.477] × 30, where Y is the absorbance of the sample.

## 4. Results and Analysis

### 4.1. Identification and Physicochemical Characterization of GlHSF Genes

Based on the screening results from hmmsearch and BLASTP, the overlapping candidate gene sequences identified by both methods were selected. A total of eight *HSF* genes were identified from the *G. lucidum* genome and were designated as *GlHSF1* to *GlHSF8*. As shown in Table 2, the deduced amino acid sequences ranged from 247 to 1352 residues in length. The corresponding molecular weights ranged from 27,307.1 Da to 147,221.35 Da, and the theoretical isoelectric points (pIs) ranged from 5.24 to 10.62. The instability index of all GlHSF proteins was greater than 40, indicating that they are all unstable hydrophilic proteins. Subcellular localization prediction suggested that most GlHSF proteins are localized to the cytoplasm and nucleus, with some possibly localized to mitochondria and a few potentially localized to the plasma membrane.

### 4.2. Chromosomal Localization and Phylogenetic Analysis of GlHSF Genes

**Figure 1:**
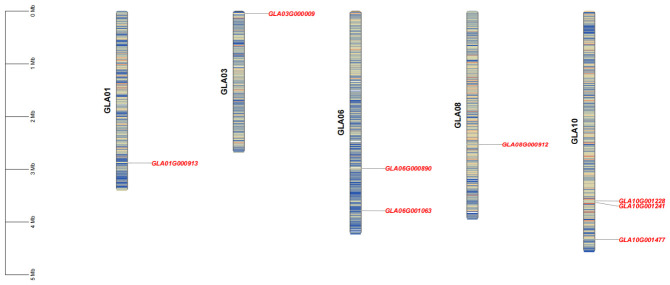
The chromosomal localization of *HSF* gene family members in *Ganoderma lucidum*. Eight *GlHSF* genes were mapped to five chromosomes (GLA01, GLA03, GLA06, GLA08, and GLA10). The vertical scale bar on the left indicates the chromosome length in megabases (Mb). Gene names and their corresponding positions are labeled alongside each chromosome.

Based on the chromosomal localization map of *GlHSF* genes (Figure 1), the eight *GlHSF* family members were found to be distributed across five different chromosomes. Chromosomes GLA01, GLA03, and GLA08 each contained only one *HSF* gene, while chromosome GLA06 harbored two *GlHSF* genes. Chromosome GLA10 contained the highest number of *HSF* genes, with three *GlHSF* family members.

**Figure 2:**
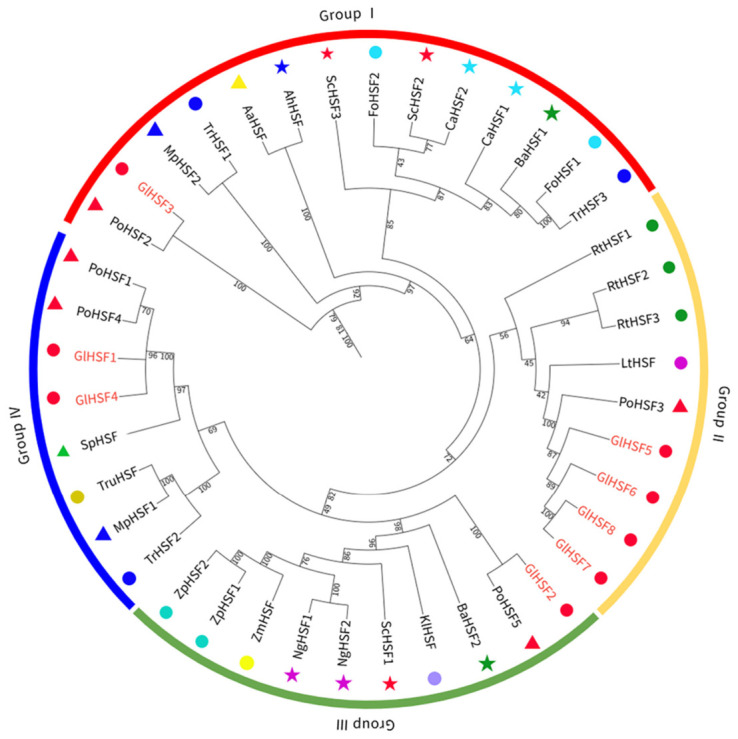
A phylogenetic analysis of *HSF* gene family members in *Ganoderma lucidum* and other fungi. The phylogenetic tree was constructed using the maximum likelihood method with 1000 bootstrap replicates in IQ-TREE. The tree was rooted and divided into four major groups (I–IV), with groups indicated by colored arcs. *Po*. *Pleurotus ostreatus*; *Sc*. *Saccharomyces cerevisiae*; *Ca*. *Candida albicans*; *Sp*. *Schizosaccharomyces pombe*; *Kl*. *Kluyveromyces lactis*; *Mp*. *Monascus purpureus*; *Tr*. *Trichophyton rubrum*; *Ba*. *Blastomyces adeninivorans*; *Zp*. *Zygosaccharomyces parabailii*; *Ng*. *Nakaseomyces glabratus*; *Lt*. *Lobosporangium transversale*; *Rt*. *Rhodotorula toruloides*; *Ah*. *Aspergillus homomorphus*; *Aa*. *Aspergillus awamori*; *Fo*. *Fusicolla oligoseptata*; *Zm*. *Zygosaccharomyces mellis*; *Tru*. *Talaromyces rugulosus*.

To elucidate the evolutionary characteristics of the *GlHSF* gene family, multiple sequence alignment was performed on 41 *HSF* genes, including eight from *G. lucidum* (*GlHSF1*–*GlHSF8*) and 33 from other fungal species such as *P. ostreatus*, *S. cerevisiae*, *and S. pombe*. A phylogenetic tree was subsequently constructed (Figure 2). Based on the topological structure of the tree, the 41 *HSF* genes were classified into four major groups (Groups I–IV).

Group I and Group III each contained one *GlHSF* gene, *GlHSF3* and *GlHSF2*, respectively. Both genes exhibited close homology with *HSF* genes from *P. ostreatus* (*PoHSF*), suggesting that these genes are evolutionarily conserved. Group II contained four *GlHSF* genes (*GlHSF5*–*GlHSF8*), which formed a highly clustered evolutionary branch, indicating close phylogenetic relationships among them. This branch was evolutionarily close to *PoHSF3* but distant from *RtHSF* of *Rhodotorula toruloides*. Group IV contained two *GlHSF* genes (*GlHSF1* and *GlHSF4*), which also clustered with *PoHSF* genes but were evolutionarily distant from *TrHSF* of *Trichophyton rubrum*.

### 4.3. Sequence Analysis and Protein Structure Prediction of GlHSF Genes

**Figure 3:**
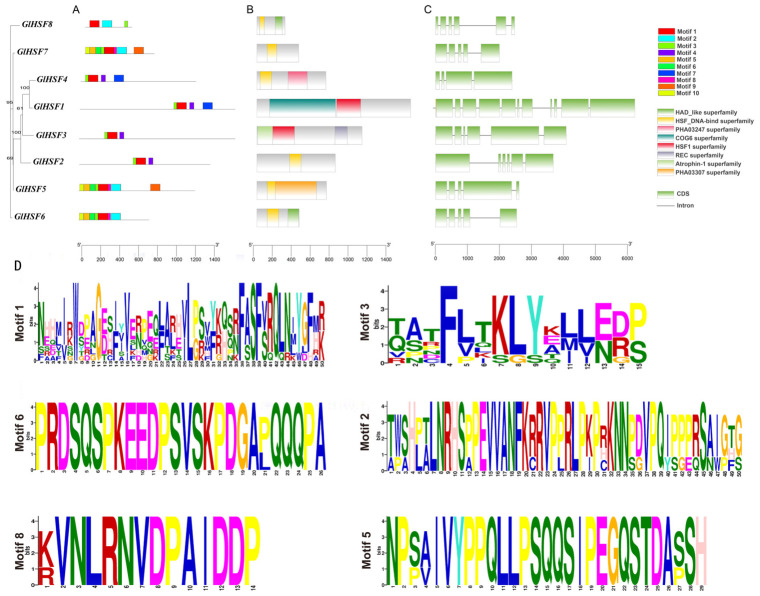
Structural characterization of HSF family members in *Ganoderma lucidum*. (**A**) Distribution of 10 conserved motifs in GlHSF proteins, as identified by MEME suite with number of motifs set to 10. (**B**) Distribution of conserved functional domains in GlHSF proteins. (**C**) Exon–intron structure of *GlHSF* genes, analyzed by GSDS 2.0 online tool. Green boxes represent coding sequences (CDSs), and black lines represent introns. (**D**) Sequence logos of six major conserved motifs in GlHSF proteins. Height of each stack reflects level of sequence conservation at that position.

Based on the MEME motif analysis (Figure 3A), all eight *GlHSF* members contained Motif 1 and Motif 3, suggesting that these two motifs are critical for *GlHSF* function. These motifs are likely associated with the DNA-binding domain or the oligomerization domain, which are essential for target sequence binding and for the monomer-to-trimer transition required for functional activation, respectively. Among the remaining motifs, Motif 2, Motif 6, Motif 8, and Motif 5 were present in four, three, three, and two *GlHSF* members, respectively. *GlHSF1*, *GlHSF2*, *GlHSF3*, and *GlHSF4* shared similar motif compositions. Similarly, *GlHSF5*, *GlHSF6*, and *GlHSF7* exhibited comparable motif distributions, consistent with their close phylogenetic relationships, indicating high structural similarity among them. *GlHSF8* contained the fewest motifs but retained two motifs common to all *GlHSF* members. Among these, Motif 2 appears critical for its function, maintaining its close evolutionary relationship with the other members. Sequence logo analysis (Figure 3D) revealed that Motif 2 and Motif 6 are highly conserved, suggesting that they play key functional roles in *HSF* genes. Genes containing these two motifs exhibited closer phylogenetic relationships. As shown in Figure 3C, the eight *GlHSF* genes contained 3–11 introns and 4–11 coding sequences (CDSs), classifying them as intron-rich genes among fungi. This suggests a high level of structural complexity within this gene family. Furthermore, all eight GlHSF proteins contained the hallmark conserved domains of the *HSF* gene family, namely the HSF_DNA-bind superfamily and the HSF1 superfamily (Figure 3B). This further confirms their identity as heat shock transcription factor family genes in *G. lucidum*.

**Figure 4:**
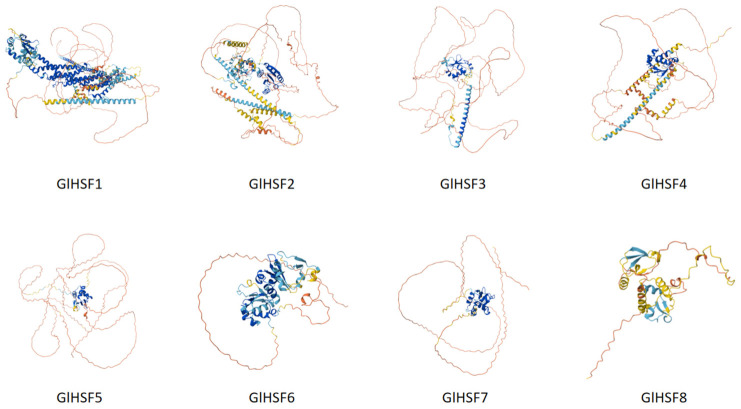
The tertiary structure prediction of the *Ganoderma lucidum* HSF family. The eight GlHSF proteins (*GlHSF1*–*GlHSF8*) exhibit conserved folding patterns, with α-helices (blue), β-sheets (yellow), and random coils (red) shown. The conserved DBD is highlighted, revealing its structural conservation across all members.

Based on the three-dimensional (3D) protein structure predictions obtained via the AlphaFold 3 online platform (Figure 4), the structural predictions for the blue and yellow regions exhibit higher confidence. As previously demonstrated, the DNA-binding domain of HSF proteins is characterized by a winged helix–turn–helix (wHTH) motif. Consistently, all eight predicted GlHSF structures conformed to this description, confirming that this motif serves as the critical functional region enabling the specific recognition of target DNA sequences.

**Figure 5:**
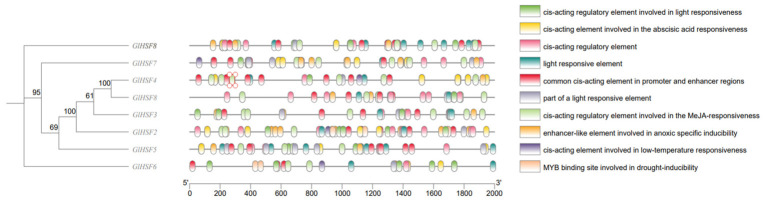
The 2000 bp promoter sequences of eight *GlHSF* genes were submitted to the PlantCARE database for cis-element prediction. Different colored boxes represent distinct cis-acting elements: light responsiveness (light green), abscisic acid responsiveness (yellow), regulatory element (pink), light-responsive element (cyan), common elements in promoter and enhancer regions (red), part of a light-responsive element (gray), MeJA responsiveness (pale green), enhancer-like element involved in anoxic specific inducibility (orange), low-temperature responsiveness (purple), and MYB binding sites involved in drought inducibility (beige). The positions of the 5′ and 3′ ends of the promoter sequences are indicated, and the scale bar represents nucleotide length.

To explore the potential transcriptional regulation of *GlHSF* genes, the 2000 bp upstream promoter regions were analyzed for cis-acting elements (Figure 5). As a highly evolutionarily conserved family of transcription factors, HSFs exhibit a high degree of homology in their regulatory elements across plants and fungi; therefore, the HSF regulatory elements in the PlantCARE database are also relevant for *G. lucidum*. The upstream regions of *GlHSF* promoters contain 10 cis-acting elements, which can be classified into four categories: abiotic stress-responsive, hormone-responsive, light-responsive, and general/structural regulatory elements. Among these, three were associated with light responsiveness, suggesting that the *GlHSF* family mediates the regulation of light signals on *G. lucidum* growth or bioactive compounds. Three are general or basic regulatory elements that maintain baseline transcription efficiency and participate in enhancer or hypoxia regulation. Two are related to hormone or signaling molecule responses, specifically abscisic acid (ABA) and methyl jasmonate (MeJA) responsiveness, which correspond to the *HSF* family’s role in counteracting temperature stress. Two are associated with abiotic stress responses, specifically low-temperature and drought stress, and are present in all eight *GlHSF* genes, suggesting that *GlHSF* may confer potential drought and cold tolerance. In summary, these results suggest that the eight *GlHSF* genes may be involved in *G. lucidum* growth, metabolism, and temperature stress resistance.

### 4.4. Expression Profile Analysis of GlHSF Genes at Different Developmental Stages of Ganoderma lucidum

**Figure 6:**
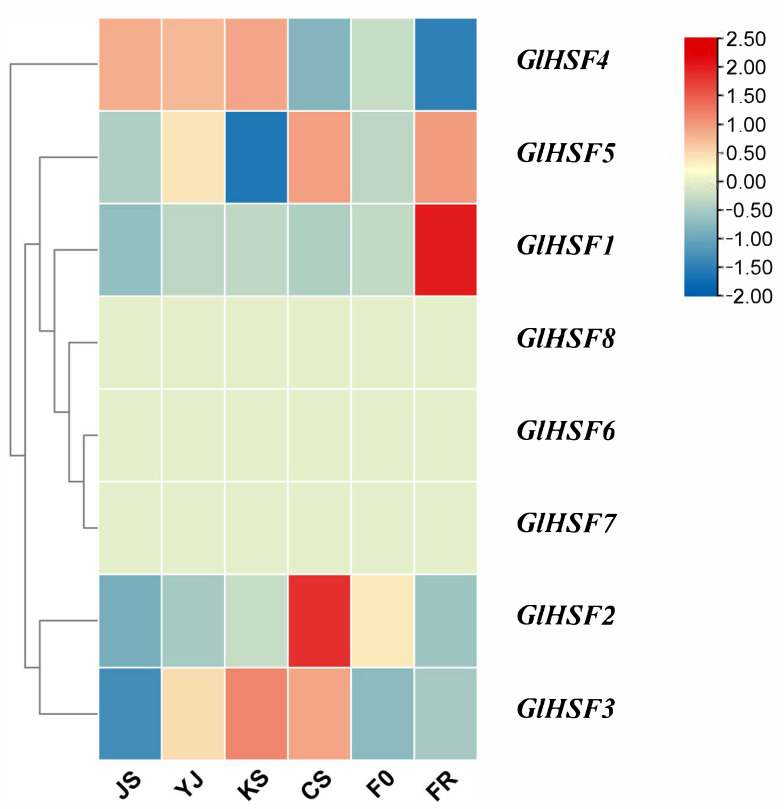
Heatmap of *HSF* gene expression in *Ganoderma lucidum* at different growth stages. Data represent three independent biological replicates, with red indicating high expression and blue indicating low expression. JS, mycelium; YJ, primordium; KS, cap opening; CS, maturity; F0, fruiting body; FR, spore powder.

The gene expression heatmap (Figure 6) shows gene expression levels in *G. lucidum* under normal growth conditions at 28 °C without temperature stress. Through cluster analysis, we found that the expression levels of *GlHSF8*, *GlHSF6*, and *GlHSF7* were nearly zero across all six growth stages, suggesting that the expression of these three *GlHSF* genes is associated with extreme temperature stress. In contrast, *GlHSF1* showed low expression levels during the first five stages but exhibited a sudden surge in expression during the spore powder stage. This suggests a potential association with spore formation. Given that the spore powder stage is the most stress-sensitive and requires the greatest stress resistance in the life cycle of *G. lucidum*, *GlHSF1* may play a protective role during spore formation.

### 4.5. Expression Analysis of GlHSF and Key Mva Pathway Enzyme Genes in Response to Temperature Stress by qRT-PCR

To investigate the response of *GlHSF* genes to temperature stress and their possible role in ganoderic acid biosynthesis, mycelia were treated at 14 °C and 42 °C, and gene expression was analyzed by qRT-PCR. *GlHSF1*, *GlHSF6*, *GlHSF7,* and *GlHSF8* had very low basal expression in mycelia (FPKM < 1) and showed no significant stress-induced changes in preliminary assays. Thus, only *GlHSF2*, *GlHSF3*, *GlHSF4*, and *GlHSF5*, which displayed detectable basal expression and clear stress responsiveness, were selected for the further analysis of their expression dynamics and potential link to the MVA pathway.

**Figure 7:**
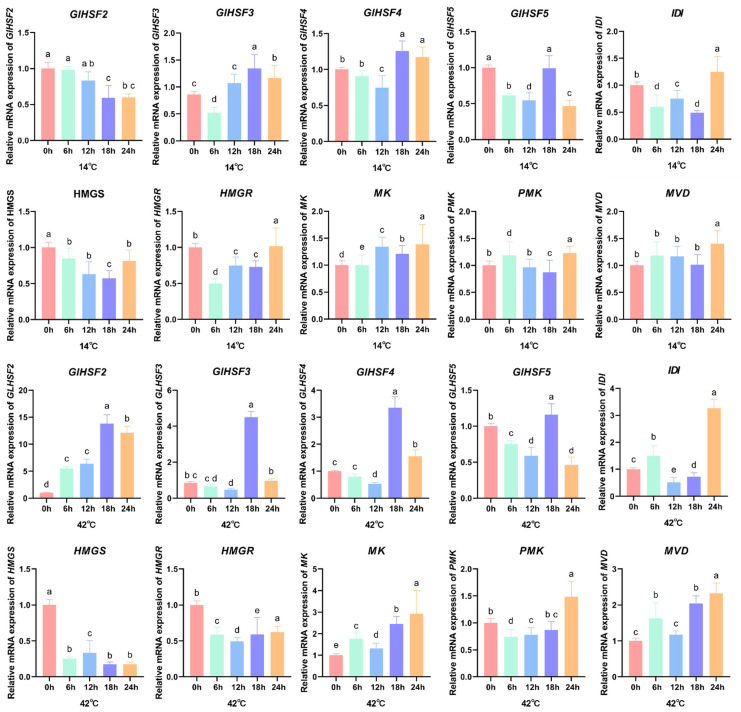
The relative expression levels of *GlHSF* genes and key mevalonate (MVA) pathway genes in *Ganoderma lucidum* under different temperature treatments. The expression levels of four *GlHSF* genes (*GlHSF2*, *GlHSF3*, *GlHSF4*, and *GlHSF5*) and six MVA pathway genes (*HMGS*, *HMGR*, *MK*, *PMK*, *MVD*, and *IDI*) were analyzed by qRT-PCR at 0, 6, 12, 18, and 24 h after exposure to 14 °C and 42 °C. Relative expression levels were calculated using the 2^−ΔΔCt^ method with 18S as the internal reference gene. Bars represent the mean ± standard deviation (SD) of three biological replicates. Different lowercase letters (a, b, c, d, e) above the bars indicate statistically significant differences between groups (one-way ANOVA, Tukey’s test, *p* < 0.05). For 42 °C, *GlHSF2* (F (4, 10) = 80.02, *p* < 0.0001); *GlHSF3* (F (4, 10) = 319.6, *p* < 0.0001); *GlHSF4* (F (4, 10) = 83.09, *p* < 0.0001); *GlHSF5* (F (4, 10) = 23.60, *p* < 0.0001); *HMGS* (F (4, 10) = 49.53, *p* < 0.0001); *HMGR* (F (4, 10) = 7.434, *p* = 0.0048); *MK* (F (4, 10) = 6.643, *p* = 0.0071); *IDI* (F (4, 10) = 23.49, *p* < 0.0001); *PMK* (F (4, 10) = 9.260, *p* = 0.0021); and *MVD* (F (4, 10) = 14.59, *p* = 0.0004).

As shown in Figure 7, under low-temperature stress (14 °C), the expression levels of *GlHSF* genes and key enzyme genes in the MVA pathway showed relatively minor changes, with relative expression levels ranging from 0.5- to 1.5-fold. In contrast, under high-temperature stress (42 °C), the relative expression levels of *GlHSF2*, *GlHSF3*, *GlHSF4*, and *GlHSF5* all peaked at 18 h. Notably, *GlHSF2* exhibited the most pronounced response, remained highly expressed from 6 to 24 h, and showed an approximately 13-fold upregulation at 18 h, suggesting that it is a candidate core factor in the heat stress response under the conditions tested. The downstream MVA pathway genes *IDI*, *PMK*, and *MVD* were significantly upregulated at 24 h, whereas the upstream rate-limiting enzyme genes *HMGR* and *HMGS* remained continuously suppressed throughout the stress period.

### 4.6. Determination of Total Triterpenoid Content in Ganoderma lucidum Mycelia Under Temperature Stress

**Figure 8:**
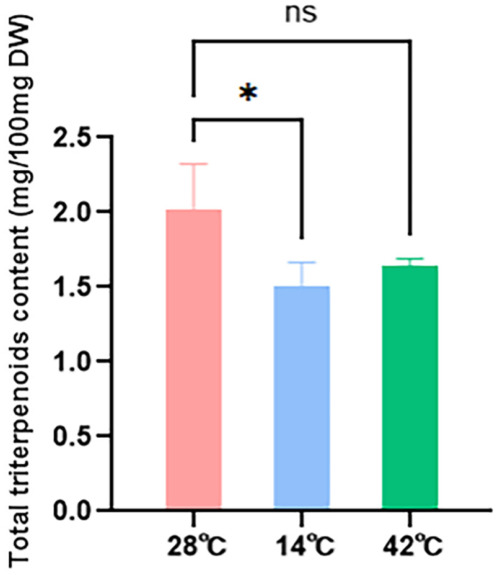
Total triterpenoid content graph of strain under temperature stress. Total triterpenoid content in *Ganoderma lucidum* mycelia under different temperature stresses. Total triterpenoid content was measured at 28 °C (control), 14 °C (low temperature), and 42 °C (high temperature). Data are presented as the mean ± standard deviation (SD) of three biological replicates. The asterisk (*) indicates a significant difference compared to the control (28 °C) at *p* < 0.05, while “ns” denotes no significant difference, as determined by the one-way ANOVA followed by Tukey’s post hoc test. The content is expressed in milligrams per 100 milligrams of dry weight (mg/100 mg DW).

A one-way ANOVA revealed a statistically significant main effect of temperature stress on total triterpenoid content in *G. lucidum* mycelia (F (2,6) = 5.334, *p* = 0.0466), followed by Tukey’s post hoc test for pairwise comparisons (Figure 8). The total triterpenoid content in *G. lucidum* mycelia varied to different degrees under different temperature stresses. Compared with the control group (approximately 2.0 mg/100 mg DW), cold stress significantly reduced total triterpenoid content to approximately 1.5 mg/100 mg DW (*p* < 0.05). Under heat stress, the content decreased to approximately 1.6 mg/100 mg DW, but this difference was not significant (*p* > 0.05). These results indicate that both low-temperature and high-temperature stress inhibit triterpenoid synthesis, with the inhibitory effect of cold stress reaching a significant level.

Under cold stress, the expression of most *GlHSF* genes showed an overall downward trend, particularly *GlHSF2* and *GlHSF5*. The expression levels of genes in the MVA pathway, such as *HMGR* and *HMGS*, also decreased. However, there were no significant differences in the expression of some key enzyme genes in the MVA pathway. This trend is consistent with the significant reduction in total triterpene content observed under cold stress. In contrast, under high-temperature stress, although the expression of most *GlHSF* genes and some key enzyme genes in the MVA pathway increased significantly, the expression of the gene encoding *HMGR*—the most critical rate-limiting enzyme in the MVA pathway—decreased significantly, thereby inhibiting the MVA pathway from the upstream. Although *HMGR* expression was significantly suppressed, limiting the upstream carbon flux into the triterpene synthesis pathway, the activation of *GlHSF* simultaneously promoted the expression of downstream genes such as *IDI*, *MK*, and *MVD*. This “upstream–downstream decoupling” regulatory pattern partially mitigated the synthetic defects caused by *HMGR* downregulation, resulting in a slight decrease in total triterpene content but no significant difference.

## 5. Discussion

In this study, eight HSF family members were systematically identified in the *G. lucidum* genome. Similarly, only seven HSF family members have been identified in *Flammulina filiformis* [58]. Although this number is higher than that found in *Saccharomyces* species, it remains considerably lower than that in plants, which possess much larger HSF families—for example, 52 in soybean and 21 in *Arabidopsis thaliana* [12]. Ascomycete fungi such as *Beauveria bassiana* [59] and *Candida albicans* [60] typically harbor three HSF members (*HSF1*, *SKN7*, and *SFL1*), which appear to be a relatively common set of HSFs across the fungal kingdom. Phylogenetic analysis placed GlHSF proteins close to those of *P. ostreatus*, indicating the evolutionary conservation of the HSF family within Agaricomycetes. Four members (*GlHSF5*–*GlHSF8*) formed a tightly clustered branch (Group II), suggesting a possible lineage-specific expansion via gene duplication. Chromosomal localization further supported this: the eight genes are distributed across five chromosomes, with three members on GLA10 (*GlHSF1*, *GlHSF2*, and *GlHSF4*). Notably, *GlHSF1* and *GlHSF4* are closely linked on GLA10 and belong to the same phylogenetic group (Group IV), pointing to a tandem duplication event. In contrast, *GlHSF2* resides on the same chromosome but in a different group (Group III), implying distinct evolutionary origins. Likewise, *GlHSF7* and *GlHSF8* on GLA06 are phylogenetically close (Group II) but not tightly linked, suggesting an older duplication followed by intrachromosomal rearrangement. These observations align with documented cases of local gene duplications driving transcription factor family expansion in fungi [61,62].

The eight *GlHSF* genes contain 3–11 introns, classifying them as intron-rich compared to many other fungal genes. In fungi, a reduction in intron number is generally an evolutionary trend that enhances transcription and splicing efficiency, thereby enabling faster stress responses [63,64]. Therefore, the high intron load in *GlHSF* genes may lead to longer splicing processing times, potentially contributing to a relatively slower transcriptional response—a hypothesis that could be tested in future time-course experiments. Fungi contain various types of cis-acting elements, such as promoters, enhancers, and silencers [65], all of which participate in the regulation of fungal development and metabolism. Promoter analysis (2 kb upstream) revealed 10 cis-acting elements, classified into four categories: abiotic stress-responsive (low-temperature, drought), hormone-responsive (ABA, MeJA), light-responsive, and general/structural elements. The presence of ABA and MeJA response elements in *GlHSF* promoters is noteworthy, as these hormones are known to mediate temperature stress adaptation in plants and fungi [66,67,68,69,70]. For example, ABA is involved in cold and drought signaling, while MeJA enhances cold tolerance via Ca^2+^ signaling and protects photosystem II under heat stress. These findings suggest that *GlHSF* expression may be modulated by upstream hormonal signals, though direct evidence is required.

Transcriptomic profiling across six stages showed that *GlHSF6*, *GlHSF7,* and *GlHSF8* were barely expressed under normal conditions, while *GlHSF1* peaked specifically at the spore powder stage, suggesting a possible role in spore formation. Under cold stress (14 °C), qRT-PCR detected only minor changes in the expression of *GlHSF* and MVA pathway genes (0.5- to 1.5-fold). In contrast, heat stress (42 °C) induced a strong upregulation of *GlHSF2*, *GlHSF3*, *GlHSF4*, and *GlHSF5*, all peaking at 18 h. Among them, *GlHSF2* showed the most pronounced response, remaining highly expressed from 6 to 24 h and reaching an approximately 13-fold increase at 18 h. A clear temporal cascade was observed: the peak of *GlHSF* expression (18 h) preceded the upregulation of downstream MVA pathway genes *IDI*, *PMK*, and *MVD* (24 h), whereas the upstream rate-limiting enzyme genes *HMGR* and *HMGS* remained continuously suppressed throughout the stress period. Consistent with this expression pattern, total triterpenoid content decreased slightly but not significantly under heat stress, whereas cold stress caused a significant reduction.

**Figure 9:**
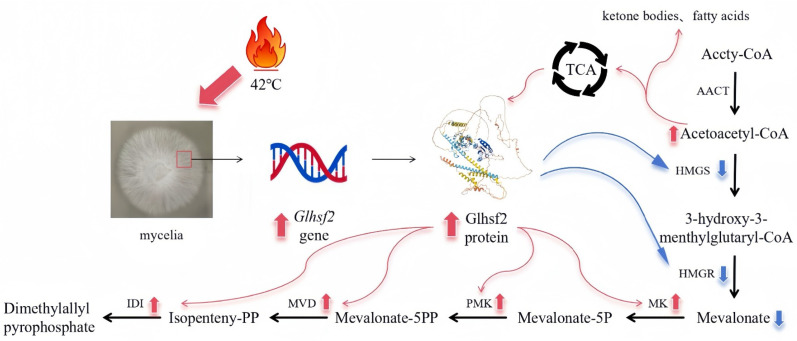
A hypothetical model illustrating the regulatory role of *GlHSF2* in the MVA pathway under heat stress. Upon heat stress, *GlHSF2* expression is upregulated, and the GlHSF2 protein is translated. *GlHSF2* then suppresses the upstream rate-limiting enzymes *HMGR* and *HMGS* while simultaneously activating the downstream genes *IDI*, *PMK*, and *MVD*. This redirection of carbon flux leads to increased flow into the tricarboxylic acid (TCA) cycle and fatty acid biosynthesis, thereby providing energy for protein synthesis, cellular repair under heat stress, and basic cellular activities. Meanwhile, the activation of downstream *MVA* genes ensures the sustained supply of precursors for basal secondary metabolism.

One of the core findings of this study is the strong response of *GlHSF2* under heat stress and its temporal correlation with the expression of downstream genes in the MVA pathway. As the most important rate-limiting enzyme known in the MVA pathway, the expression of *HMGR* is continuously suppressed under heat stress, which contrasts sharply with the activation of downstream genes such as *IDI*. Under high-salt stress, the MVA pathway is upregulated at the transcriptional level, and key enzymes, including *ACAT*, *HMGR* and *IDI*, are significantly induced, thereby promoting the flow of carbon to squalene synthesis. Despite the downregulation of SQS, squalene still accumulated and increased, which was attributed to the increased supply of precursors and the reduced flux to the downstream sterol pathway [71]. Under heat stress, this dynamic change shows a significant time cascade effect: the peak expression of *GlHSF* (18 h) occurs earlier than the upregulation of the downstream MVA gene (24 h), while the upstream gene remains in an inhibited state all the time. From this, it can be inferred that the mechanism logic is similar under high-temperature stress. We hypothesize that *GlHSF2* may differentially regulate the MVA pathway under thermal stress; that is, while inhibiting the upstream *HMGR* and *HMGS*, it activates the downstream *IDI*, *PMK*, and *MVD* to maintain the potential ability of downstream precursors (such as IPP and DMAPP) to transform. This is a speculative regulatory (Figure 9) approach by which *G. lucidum* prioritizes survival under heat stress (inhibiting major metabolic pathways) while maintaining basic secondary metabolism (activating terminal enzymes).

However, this study is mainly based on bioinformatic prediction and correlation analysis. If the function of *GlHSF* is to be further verified, future research should mainly focus on using yeast single-hybrid (Y1H) or electrophoretic mobility shift assay (EMSA) and other experiments to verify whether *GlHSF2* specifically binds to HSF binding sites in the promoter regions of key enzyme genes in the MVA pathway, such as *IDI* and *MK*, and whether it can directly participate in regulation. And overexpression or gene knockout techniques could be used to modify the strains, and the growth of the modified strains under heat stress, the expression of key genes, and the accumulation of metabolites could be observed. The quantification of individual ganoderic acids by LC-MS will help identify the precise metabolic nodes modulated by GlHSF. In addition, in combination with the cis-element of the *GlHSF* promoter region, taking ABA and MeJA response elements as examples, the existence of exogenous hormones or their inhibitors was verified by observing the changes in gene expression after adding them to the mycelial medium, and their impact on subsequent regulation was investigated. Expanding and integrating these contents will facilitate the analysis of the functions and regulatory modalities of *GlHSF* and help increase the accumulation of secondary metabolites such as ganoderic acid.

## 6. Conclusions

In summary, eight *GlHSF* genes were identified in the *G. lucidum* genome, and their structural features, expression patterns under temperature stress, and potential regulatory functions in the MVA pathway were comprehensively analyzed. Among these, *GlHSF2* displayed a marked response to heat stress and is regarded as a candidate core factor in the heat stress response, warranting further functional investigation. The eight *GlHSF* genes play distinct or synergistic potential roles in regulating growth, development, secondary metabolite biosynthesis, and stress responses across the six developmental stages of *G. lucidum*. These findings provide a theoretical foundation for further functional studies of genes in *G. lucidum*.

## Figures and Tables

**Table 1:** Primers of genes in qRT-PCR.

Gene Name	Forward Primer (5′–3′)	Reverse Primer (5′–3′)
*Gl* *HSF* *2*	ACATCAAGCGTAAGGTGCC	TGGTAGTTGGTCTCAAGGGA
*Gl* *HSF* *3*	GCCAGCACTCGTATCAACAC	CGCAGCACTAACGCCAT
*Gl* *HSF* *4*	GCCCCAGCACTCTTTCAT	CGGCTTCGGACCATTTTA
*Gl* *HSF* *5*	AGCCCGCTGCTATCAACA	GCGAGTAGTCATGCGAAAAGT
*IDI*	CCAGTTCCTTCTCCGGTGTG	TCGCGGATCTCGTTGACATT
*HMGS*	GACGTTGGTATCCTCGCCAT	CCGATGGTGTACTTGCCCTT
*HMGR*	TGGAGCCTATTACCCCCGAA	GGCGATCTTTCCAGTTTGGC
*MK*	GTCGGGAGTGGTTTGTCAGT	GCGAAATGCACCGACACTTT
*PMK*	CTCGGCCACAAACAACAAGT	GATGTCCAAGCCATGACCGA
*MVD*	TAAAGTACTGGGGCAAGCGG	TTAATGCATGTTGCGAGCCG
*18S*	TATCGAGTTCTGACTGGGTTGT	ATCCGTTGCTGAAAGTTGTAT

**Table 2:** Physicochemical properties of *HSF* gene family members in *Ganoderma lucidum*.

Gene Name	Gene ID	Amino Acid Length	Molecular Weight/Da	pI	Instability Index	Subcellular Localization
*GlHSF1*	GLA10G001241	1352	147,221.35	5.53	48.84	nucl, cyto_nucl, mito, cyto
*GlHSF2*	GLA10G001477	925	99,925.4	6.15	62.89	nucl
*GlHSF3*	GLA01G000913	690	74,841.57	7.33	67.03	nucl
*GlHSF4*	GLA10G001228	607	65,941.35	5.24	57.87	nucl, cyto_nucl, cyto
*GlHSF5*	GLA08G000912	612	65,926.01	8.72	70.47	nucl, cyto_nucl, cyto, plas
*GlHSF6*	GLA03G000009	371	40,850.9	7.07	68.43	nucl, cyto_nucl, mito, cyto
*GlHSF7*	GLA06G000890	367	39,904.21	9.4	81.01	nucl, cyto_nucl, mito, cyto
*GlHSF8*	GLA06G001063	247	27,307.1	10.62	56.79	mito, plas, nucl, cyto

## Data Availability

The data presented in this study are largely available within the article and its Supplementary Materials. The raw transcriptome sequencing datasets generated during the current study are not publicly available due to a combination of internal laboratory confidentiality policies and ongoing commercial collaboration agreements. Requests for access to the restricted datasets should be directed to the corresponding author and will be considered on a case-by-case basis, subject to the approval of the involved commercial partners and the institutional review board.

## Supplementary Materials

The following supporting information can be downloaded at https://www.mdpi.com/article/10.3390/genes17040473/s1, Figure S1: Figures of the six distinct developmental stages of *Ganoderma lucidum*.
