# Supplementary material for: The Genome-Wide Identification and Expression Profiling of the HSF Gene Family in Ganoderma lucidum Under Temperature Stress
Source: Genes (Basel). 2026 Apr 17;17(4):473. doi: 10.3390/genes17040473 (PMC13116305; doi:10.3390/genes17040473)
Supplement: Supplementary file 1 [file genes-17-00473-s001.zip › genes-4254037-supplementary.pdf]

Supplementary Figures

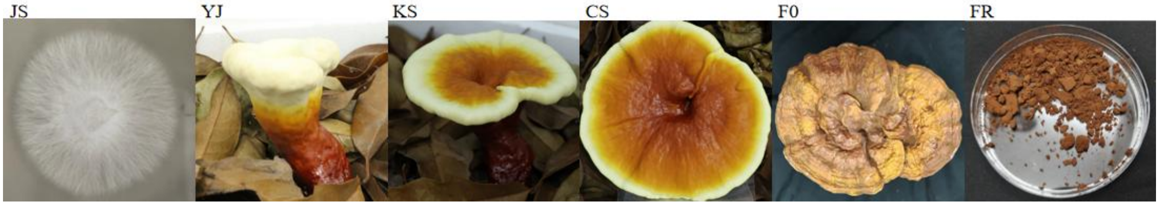

Supplementary Figure S1. Figures of the six distinct developmental stages of *Ganoderma lucidum*: (A) JS, mycelium; (B) YJ, primordium; (C) KS, cap opening; (D) CS, maturity; (E) F0, fruiting body; (F) FR, spore powder.
